# Impact of growth hormone on IVF/ICSI outcomes and endometrial receptivity of patients undergoing GnRH antagonist protocol with fresh embryo transfer: a pilot study

**DOI:** 10.3389/fendo.2023.1225121

**Published:** 2023-08-31

**Authors:** Yifan Chu, Luyao Wang, Jiaxin Xie, Shulin Yang, Si Liu, Dan Hu, Jing Yue

**Affiliations:** Reproductive Medicine Center, Tongji Hospital, Tongji Medical College, Huazhong University of Science and Technology, Wuhan, China

**Keywords:** growth hormone, IVF/ICSI, endometrial receptivity, GnRH antagonist, fresh embryo transfer

## Abstract

**Introduction:**

Gonadotropin-releasing hormone antagonist (GnRH-ant) protocol is widely used in the world for controlled ovarian hyperstimulation (COH). However, previous studies have shown that pregnancy outcomes of fresh embryo transfer with GnRH-ant protocol are not ideal. Current studies have demonstrated the value of growth hormone (GH) in improving the pregnancy outcome of elderly women and patients with diminished ovarian reserve, but no prospective studies have confirmed the efficacy of GH in fresh embryo transfer with GnRH-ant protocol, and its potential mechanism is still unclear. This study intends to evaluate the impact of GH on IVF/ICSI outcomes and endometrial receptivity of patients undergoing GnRH-ant protocol with fresh embryo transfer, and preliminarily explore the possible mechanism.

**Methods:**

We designed a randomized controlled trial of 120 infertile patients with normal ovarian response (NOR) who will undergo IVF/ICSI from April 2023 to April 2025, at Department of Reproductive Medicine, Tongji Hospital, Tongji Medical College, Huazhong University of Science and Technology. The patients will be divided into the depot gonadotropin-releasing hormone agonist (GnRH-a) protocol group, GnRH-ant protocol control group, and GnRH-ant protocol plus GH intervention group at a ratio of 1:1:1 by block randomization design. Patients will be followed on enrollment day, trigger day, embryo transfer day, 7 days after oocytes pick-up, 15 days after embryo transfer, 28 days after embryo transfer, and 12 weeks of gestation. The primary outcome is the ongoing pregnancy rate. Secondary outcomes include the gonadotropin dosage, duration of COH, endometrial thickness and pattern, luteinizing hormone, estradiol, progesterone level on trigger day, numbers of retrieved oocytes, high-quality embryo rate, biochemical pregnancy rate, clinical pregnancy rate, implantation rate, ectopic pregnancy rate, early miscarriage rate, multiple pregnancy rate and incidence of moderate and severe ovarian hyperstimulation syndrome. The endometrium of certain patients will be collected and tested for endometrial receptivity.

**Ethics and dissemination:**

The study was approved by the Ethics Committee of Tongji Hospital, Tongji Medical College, Huazhong University of Science and Technology [approval number: TJ-IRB20230236; approval date: February 10, 2023]. The research results will be presented at scientific/medical conferences and published in academic journals.

**Clinical trial registration:**

Chinese Clinical Trial Registry; identifier: ChiCTR2300069397.

## 1 Introduction

With the rapid development of assisted reproductive technology (ART), seeking an efficient and safe ovarian stimulation protocol is the main pursuit of doctors worldwide. Gonadotropin-releasing hormone agonist (GnRH-a) protocol, gonadotropin-releasing hormone antagonist (GnRH-ant) protocol, progesterone primed ovarian stimulation (PPOS) protocol, and minimal ovarian stimulation protocol are commonly used worldwide. The GnRH-ant protocol has received global attention for its simplified treatment course, shortened treatment period, and low incidence of ovarian hyperstimulation syndrome (OHSS) (1, 2). In 2020, the European Society of Human Reproduction and Embryology (ESHRE) published guidelines for controlled ovarian hyperstimulation (COH), indicating that GnRH-ant protocol can be used as first-line treatment for patients with high, normal, or poor ovarian response (3). Considering the safety of COH, ESHRE guidelines strongly recommended GnRH-ant protocol as a prior treatment for normal ovarian response (NOR) patients (3, 4).

For patients with NOR, the ideal therapeutic goals are to reduce the duration of COH, shorten the time to live birth and improve the pregnancy rate of fresh embryo transfer (1). However, previous studies have demonstrated that pregnancy outcomes of fresh embryo transfer with GnRH-ant protocol are not ideal. A retrospective study involving 203,302 fresh embryo transfer IVF cycles revealed a significantly higher cycle cancellation rate and a significant lower implantation rate and clinical pregnancy rate on the GnRH-ant protocol compared with those on the GnRH-a protocol (5). Another retrospective study indicated that the clinical pregnancy rate and live birth rate of patients receiving fresh embryo transfer with GnRH-ant protocol were significantly lower than those of frozen embryo transfer (6).

In recent years, how to promote the pregnancy rate of fresh embryo transfer with GnRH-ant protocol has become a research hotspot. Several studies have shown that serum luteinizing hormone (LH) levels, progesterone levels, trigger methods, and the method of GnRH antagonist usage during COH are associated with adverse pregnancy outcomes, but there is still a lot of controversy (7–13).

Growth hormone (GH) is a single-chain polypeptide hormone secreted by the anterior pituitary eosinophilic cells, consisting of about 190 amino acids. It has been more than 30 years since GH was first reported to be applied in the field of ART in 1988 (14). Current studies have shown that GH can regulate primordial follicle development, improve ovarian response to gonadotropin (Gn), improve oocyte quality, improve oocyte and granulosa cell mitochondrial function and oxidative stress state, and thus improve the pregnancy outcome of elderly women and patients with poor ovarian response (POR) (15). A meta-analysis consisting of 15 randomized controlled trials (RCTs) involving 1448 patients with POR found that GH can significantly increase the live birth rate, clinical pregnancy rate, and the number of oocytes retrieved, while significantly reducing the cycle cancellation rate and Gn dosage (16). Expert consensus on clinical diagnosis and treatment of POR patients in China points out that GH is recommended during the ART process for patients with POR, low embryo quality, thin endometrium, and recurrent implantation failure (RIF) (17). The guidelines also explicitly suggest the possibility of GH in improving endometrial receptivity.

A recent meta-analysis involving 17 RCTs found that GH supplementation may benefit patients with POR by improving endometrial thickness, clinical pregnancy rate, and live birth rate (18). Some studies have also confirmed that GH can improve pregnancy outcomes for patients with thin endometrium and RIF (19, 20). Besides, a single-center retrospective study including 80 patients who received GH treatment for POR or low fertilization rate or low blastocyst formation rate in the previous cycle found that GH can significantly increase the endometrial thickness without significant side effects or untoward reaction (21). However, no prospective study has confirmed the efficacy of GH in fresh embryo transfer with GnRH-ant protocol, and its potential mechanism has not been studied. This study intends to enroll NOR patients receiving fresh embryo transfer with GnRH-ant protocol to evaluate whether GH supplementation can improve the pregnancy outcome of fresh embryo transfer, and preliminarily explore the possible mechanism.

## 2 Methods and data analysis

### 2.1 Study design

This is a pilot study of infertile patients with NOR who will undergo *in vitro* fertilization and embryo transfer (IVF-ET) or intracytoplasmic sperm injection (ICSI) at Department of Reproductive Medicine, Tongji Hospital, Tongji Medical College, Huazhong University of Science and Technology from April 2023 to April 2025. According to the sample size estimation, 120 patients would be expected to be recruited for this study.

The primary inclusion criteria are (1) female aged 20-35; (2) menstrual cycle is between 25 and 35 days; (3) the body mass index (BMI) is between 18.5 and 25kg/m^2^; (4) basal follicle-stimulating hormone (FSH)<10 IU/mL; (5) antral follicle count (AFC) is between 7 and 15 or anti-mullerian hormone (AMH) is between 1.1 and 4.0 ng/ml; (6) IVF or ICSI for the first time; (7) the cause of infertility is male infertility, tubal infertility or unexplained infertility.

**Figure 1:**
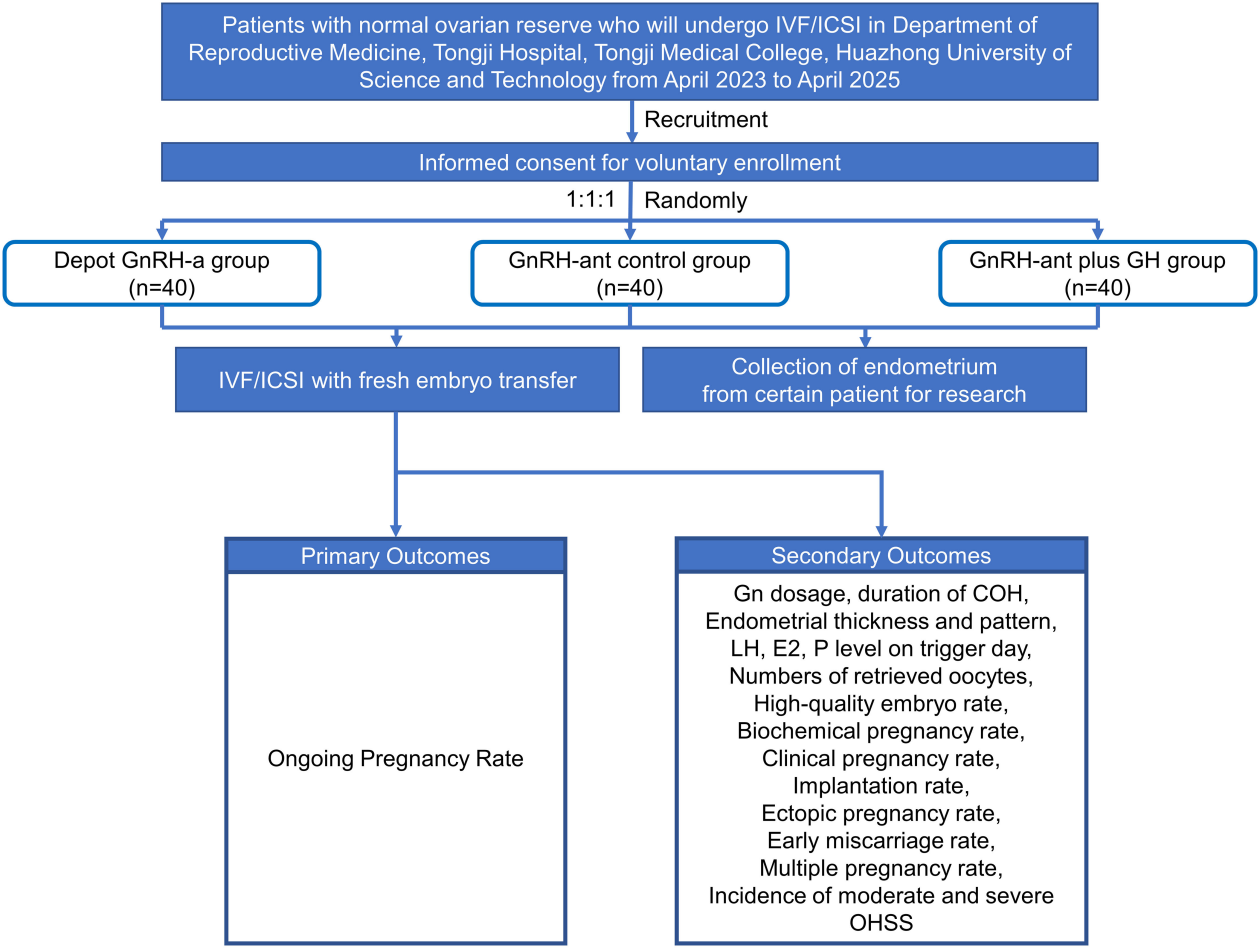
Flow chart of the study design.

The primary exclusion criteria are (1) AFC<7 or >15 and AMH<1.1ng/ml or >4ng/ml; (2) polycystic ovarian syndrome (PCOS) patient; (3) ovarian or fallopian tube diseases, such as ovarian tumor, untreated severe hydrosalpinx, endometriosis cyst, etc.; (4) abnormalities of uterus or uterine cavity, such as adenomyosis, uterine submucosal fibroids or intramural fibroids affecting uterine cavity, genital tract malformations, endometrial polyps, intrauterine adhesions, thin endometrium, etc.; (5) preimplantation genetic testing due to chromosomal abnormalities, recurrent implantation failure (RIF), recurrent spontaneous abortion (RSA), or monogenic genetic diseases, etc.; (6) ICSI due to asoospermia or extremely severe oligospermia, asthenospermia (the sperm concentration was < 1×10^6^/ml and the percentage of forward motility sperm was < 1%, and the total number of forward motility sperm after processing less than one million), specific teratospermia (e.g., round head spermia, macrocephalic spermia, headless spermia, and multiple malformations of sperm flagella) or testicular sperm aspiration (TESA) or percutaneous epididymal sperm aspiration (PESA); (7) Other system-related diseases, such as thyroid diseases, diabetes, adrenal disorders, uncontrolled and untreated chronic diseases, suffering from diseases that are not suitable for pregnancy, etc.; (8) GH contraindications, such as diabetes mellitus, severe systemic infection, active tumor, active intracranial injury, pregnancy, etc.; (9) Pretreatment of other drugs recently, such as oral contraceptives, coenzyme Q10, metformin, etc.; (10) patient in other clinical studies at the same time, or the researcher deems it inappropriate to participate in this study (**Table 1**). Participants will be divided into depot GnRH-a group, GnRH-ant control group, and GnRH-ant plus GH intervention group randomly. We will investigate whether GH affects pregnancy outcomes and endometrial receptivity in patients undergoing GnRH-ant protocol with fresh embryo transfer (**Figure 1**).

**Table 1:** The inclusion and exclusion criteria for selecting the study participants.

Primary inclusion criteria	Primary exclusion criteria
• Aged 20-35	• AFC <7 or >15 and AMH <1.1ng/ml or >4ng/ml
• Menstrual cycle 25-35 days	• PCOS
• BMI 18.5-25kg/m^2^	• Ovarian or fallopian tube diseases (e.g. ovarian tumor, untreated severe hydrosalpinx, endometriosis cyst, etc.)
• Basal FSH<10 IU/mL AFC 7-15 or AMH 1.1-4.0 ng/ml	• Abnormalities of uterus or uterine cavity (e.g. adenomyosis, uterine submucosal fibroids or intramural fibroids affecting uterine cavity, genital tract malformations, endometrial polyps, intrauterine adhesions, thin endometrium, etc.)
• IVF/ICSI for the first time	• PGT
• Male infertility or tubal infertility or unexplained infertility	• Asoospermia or extremely severe oligospermia, asthenospermia or specific teratospermia or TESA/PESA
	• Other system-related diseases (e.g. thyroid diseases, diabetes, adrenal disorders, uncontrolled and untreated chronic diseases, suffering from diseases that are not suitable for pregnancy, etc.)
	GH contraindications (e.g. diabetes mellitus, severe systemic infection, active tumor, active intracranial injury, pregnancy, etc.)
	• Recent pretreatment of other drugs (e.g. oral contraceptives, coenzyme Q_10_, metformin, etc.)
	• Patient in other clinical studies at the same time, or the researcher deems it inappropriate to participate in this study

BMI, body mass index; FSH, follicle stimulating hormone; AFC, antral follicle count; AMH, anti-mullerian hormone; IVF-ET, in vitro fertilization and embryo transfer; ICSI, intracytoplasmic sperm injection; PCOS, polycystic ovarian syndrome; PGT, preimplantation genetic testing; TESA, testicular sperm aspiration; PESA, percutaneous epididymal sperm aspiration.

### 2.2 Procedures

Patients who meet the above criteria will voluntarily decide whether to participate in this study. Informed consent would be signed by the designated doctor and the patient. A total of 120 infertile patients would be enrolled. The patients would be divided into the depot GnRH-a protocol group, GnRH-ant protocol control group, and GnRH-ant protocol plus GH intervention group at a ratio of 1:1:1 by block randomization design. Considering the open-label design of this study, both the attending nurse and the patient would be informed of the intervention method, while the embryologist and laboratory technician would be unaware of the intervention method. Participants’ baseline characteristics would be acquired from the hospital information database, including age, body mass index, primary infertility/secondary infertility; infertility duration; cause of infertility, history of fertility treatment; past medical history; history of drug allergy; vital signs; physical examination; urine hCG test; electrocardiogram; basal FSH, LH, E2, PRL, T, P, AMH, AFC; blood routine; urine routine; blood biochemistry; coagulation function; blood type; hepatitis B, syphilis, HIV examination, etc. will also be recorded.

#### 2.2.1 COH protocol

Each group received different treatments as follows:

##### 2.2.1.1 Depot GnRH-a protocol group

The patient would be given a subcutaneous injection of 3.75mg leuprorelin (Biote Pharmaceutical, Beijing, China) or 3.75mg triptorelin (Dophereline^®^, Ipsen, France/Decapeptyl^®^, Ferring, Switzerland) after endometrial thickness was determined to be <5mm with no follicle growth by transvaginal ultrasonography (TVS) on day 2 of the previous menstrual cycle. Approximately 28 days after injection, the patient would be confirmed to have reached the pituitary down-regulation position (E2 ≤30pg/ml and LH ≤2IU/L). Recombinant follicle-stimulating hormone (rFSH, Genescience Pharmaceutical, Changchun, China) would be selected for COH. The initial dose would be selected by the attending doctor according to the patient’s age, BMI, and ovarian reserve, and the dosage would be 150-225IU per day. During COH, doctors are allowed to adjust the dosage of Gn according to the patient’s ovarian response, and the maximum daily dosage would not exceed 450IU. When patients have a slow ovarian response during COH, human menopausal gonadotropin (HMG) (Lizon Pharmaceutical, Zhuhai, China) is allowed, and the dosage is determined by the attending doctor. During COH, any other adjuvant drugs are not allowed. When patients have two dominant follicles ≥18mm or three follicles ≥17mm in diameter, they would be triggered with recombinant human chorionic gonadotropin (rhCG, Ovidrel^®^, Merck Serono, Germany).

##### 2.2.1.2 GnRH-ant protocol control group

COH would be performed by a fixed GnRH-ant protocol. The patients would receive COH from day 2 or 3 of menstruation. The selection and adjustment of Gn and the treatment of slow ovarian response would be the same as those in the depot GnRH-a protocol group. During COH, any other adjuvant drugs are not allowed. On the 5th or 6th day of COH, 0.25mg cetrorelix acetate (Cetrotide ^®^, Merck Serono, Germany) or ganirelix acetate (Orgalutran^®^, Organon, USA/QingLe^®^, CHIA TAI TIANQING, Lianyungang, China) would be injected subcutaneously into the trigger every day. rHCG (Ovidrel^®^, Merck Serono, Germany) trigger would be used if the patient is not at high risk for OHSS. If the patient is at high risk for OHSS (trigger day E2≥5000pg/ml or ≥18 follicles with a diameter larger than 14mm), 0.2mg GnRH-a (Decapeptyl^®^, Ferring, Switzerland) or HCG 2000IU combined with 0.2mg GnRH-a (Decapeptyl^®^, Ferring, Switzerland) would be used for trigger. Other medications are at the discretion of the attending doctor.

##### 2.2.1.3 GnRH-ant protocol plus GH intervention group

Based on the GnRH-ant protocol control group, all patients will receive subcutaneous injection of 5IU GH (Genescience Pharmaceutical, Changchun, China) once a day from the beginning of COH until the trigger day.

#### 2.2.2 Oocytes pick-up and embryo transfer

Oocytes would be picked up 36-38 hours after the trigger. During the OPU, follicular fluid from dominant follicles (diameter ≥18mm, oocytes visible under microscopy) that are not contaminated by blood would be collected, centrifuged, and stored in a -80°C refrigerator. The remaining follicular fluid and follicular flushing fluid would be collected in 50ml centrifuge tubes, and granulosa cells would be separated and extracted by enzymolysis method, and stored at -80°C in the refrigerator. 16-18 hours after insemination, the embryonic pronucleus (PN) would be observed. The embryos would be observed on day 3 after insemination. High-quality embryos were defined as 7-10 cell stage embryos of 2PN origin with blastomere fragments ≤20% at day 3 after fertilization (22). For patients meeting the criteria of fresh embryo transfer, one day 3 high-quality embryo would be transferred, and all remaining embryos would be cultured to blastocyst stage and frozen. For patients who need to cancel fresh embryo transfer due to various reasons (**Table 2**), 0-1 day 3 high-quality embryos would be frozen according to the embryo condition, and the remaining embryos would be cultured to the blastocyst stage and frozen.

**Table 2:** Reasons for canceling fresh embryo transfer.

Reasons	details
• High risks of OHSS	1. E2≥5000pg/ml or ≥18 follicles (GnRH-a trigger) on the trigger day; 2. E2≥1500pg/ml; 3. ≥18 oocytes obtained; 4. 1000pg/ml≤E2 ≤ 1500pg/ml on the day after OPU but the patient had mild OHSS symptoms.
• Endometrial and fallopian tube factors	1. Hydrosalpinx and intrauterine effusion occurred during COH; 2. Endometrial thickness ≤6mm on ET day; 3. Single endometrial polyps >10mm or multiple endometrial polyps (regardless of size);
• High progesterone level	1. blood progesterone level ≥1.5ng/ml on the trigger day; 2. blood progesterone level ≥1ng/ml for three consecutive days during the COH.
• Embryo factor	No high-quality day 3 embryos for transfer
• Patients’ factor	Patients request cancellation of fresh embryo transfer

OHSS, ovarian hyperstimulation syndrome; E2, estradiol; OPU, oocytes pick-up; COH, controlled ovarian hyperstimulation.

For patients who meet the criteria for fresh embryo transfer, they would receive vaginal progesterone sustained-release gel (Crinone^®^, Merck Serrano, Germany) 90mg per day or progesterone soft capsules (Utrogestan^®^, Besin, Belgium) 200mg per day plus dydrogesterone tablets (Duphaston^®^, Abbott, USA) 10mg twice a day for luteal support from the day after the OPU. All patients would undergo ET mediated by TVS. β- hCG would be detected 14 days after ET, and pregnant patients would continue to be given luteal support. After 2 weeks, the dosage of luteal support drugs would be gradually reduced in patients with at least one intrauterine gestational sac and heart tube beating by TVS. Luteal support drugs will be discontinued at 10 weeks of gestation.

#### 2.2.3 Collection of endometrium

For patients who received the rhCG trigger and canceled fresh embryo transfer, endometrium could be collected if there were no obvious endometrial abnormality, OHSS and no elevated progesterone level. After the attending doctor communicates with the patient and fully informs the patient of relevant matters, the patient can voluntarily choose whether to take endometrial sampling.

For patients with endometrium collection who were determined to freeze whole embryos on trigger day, dydrogesterone tablets (Duphaston^®^, Abbott, USA) 10mg twice a day would be given for luteal support; For patients with endometrium collection who decide to freeze whole embryos after OPU, discontinue the use of Crinone^®^ (Merck Serrano, Germany).

Endometrium collection would be performed in all eligible patients on day 5 after OPU. A small amount of endometrial tissue would be obtained and rinsed with physiological saline. The endometrial tissue would be rapidly frozen in liquid nitrogen and stored in the refrigerator at -80°C for a long time.

#### 2.2.4 Endometrium research

For the obtained endometrium samples, the following *in vitro* studies and tests would be performed:

##### 2.2.4.1 Transcriptome analysis of endometrial tissues

RNA would be extracted from 5 endometrial tissues in each group (15 endometrial tissue samples in total) by Trizol, and eukaryotic reference transcriptome would be detected by next-generation sequence (NGS), and subsequent bioinformatics analysis would be performed by R software. The obtained results would be verified by real-time quantitative PCR (qRT-PCR).

##### 2.2.4.2 Endometrial receptivity molecular detection

qRT-PCR and Western blotting would be used to detect the expression of mRNA and protein levels of endometrial receptivity and decidualization molecules, including LIF, integrinβ3, S100P, HOXA10, SCARA5, DIO3, FoxO1, etc (23–26).

#### 2.2.5 Follow-up plan

Patients would be followed on enrollment day, trigger day, ET day, 7 days after OPU, 15 days after ET, 28 days after ET, and 12 weeks of gestation (**Table 3**).

**Table 3:** Overview of follow-up during the study.

Time	Follow-up Information
• Enrollment day	Patient randomization, collection of baseline characteristics of the participants (age, body mass index, primary infertility/secondary infertility; infertility duration; cause of infertility, history of fertility treatment; past medical history; history of drug allergy; vital signs; physical examination; urine hCG test; electrocardiogram; basal FSH, LH, E2, PRL, T, P, AMH, AFC; blood routine; urine routine; blood biochemistry; coagulation function; blood type; hepatitis B, syphilis, HIV examination, etc.).
• Trigger day	LH, E2, P levels; TVS; GH medication records; rFSH medication record; GnRH-ant medication record; HMG medication record; rhCG or GnRH-a medication record; untoward reaction record.
• ET day	Oocytes status; embryo status; luteal support medication record; number and morphological evaluation of transferred embryos; untoward reaction record.
• 7 days after OPU	Blastocysts status, collection of endometrium samples; untoward reaction record.
• 15 days after ET	serum β-hCG levels; luteal support medication record; untoward reaction record.
• 28 days after ET	TVS; luteal support medication record; untoward reaction record.
• 12 weeks of gestation	Pregnancy outcomes (ongoing pregnancy rate or early miscarriage or ectopic pregnancy); untoward reaction record.

hCG, human chorionic gonadotropin; FSH, follicle stimulating hormone; LH, luteinizing hormone; E2, estradiol; PRL, prolactin; T, testosterone; P, progesterone; AMH, anti-mullerian hormone; AFC, antral follicle count; HIV, human immunodeficiency virus; TVS, transvaginal sonography; GH, growth hormone; HMG, human menopausal gonadotropin; OPU, oocytes pick-up; ET, embryo transfer.

### 2.3 Primary outcomes

The primary outcome is the ongoing pregnancy rate (OPR). OPR was defined as a clinical pregnancy until after 12 weeks of gestation.

### 2.4 Secondary outcomes

The secondary outcomes in this study include the Gn dosage, duration of COH, endometrial thickness and pattern, LH, E2, P level on trigger day, numbers of retrieved oocytes, high-quality embryo rate, biochemical pregnancy rate, clinical pregnancy rate, implantation rate, ectopic pregnancy rate, early miscarriage rate, multiple pregnancy rate and incidence of moderate and severe OHSS. The high-quality embryo was defined as embryos with 7-10 cells of 2PN origin and blastomere fragments ≤20% on day 3 after fertilization (22). Biochemical pregnancy was defined as a serum level of β-hCG of more than 10 mIU per milliliter (27). Clinical pregnancy was determined when at least one gestational sac was observed on TVS. Early miscarriage was defined as pregnancy loss before 12 weeks of gestation.

### 2.5 Sample size calculations

According to the previous literature review, no study has reported whether GH can improve the ongoing pregnancy rate of patients undergoing fresh embryo transfer with GnRH-ant protocol. Combined with the data of our center, the ongoing pregnancy rate of patients receiving fresh embryo transfer under GnRH-ant protocol was about 51.5%, and that of patients receiving depot GnRH-a protocol was about 69.7% (unpublished), while GH could increase the ongoing pregnancy rate by about 12%. The sample size was estimated using the chi-square test with α=0.05 and 1-β=0.8, then we need about 133 people per group, considering 10% shedding, about 147 people per group are needed.

Considering that this study is a pilot study designed to initially evaluate the feasibility, safety, efficacy, and range of variation of the GH intervention. Based on the above considerations, one-third to a quarter of the estimated sample size was selected for the pilot study, 40 patients are included in each group, and 120 patients would be expected to be recruited for this study.

### 2.6 Statistical analysis

In this study, IBM SPSS Statistics V21.0 software would be used for data analysis. Measurement data would be expressed as mean ± standard error and count data would be expressed as rate (%). Kolmogorov-Smirnov would be used for the normality test and Levene would be used for the homogeneity of variance test. If the statistical data were consistent with normal distribution, analysis of variance and Kruskal-Wallis H were used to compare the differences in clinical outcomes among the three groups. Posthoc analysis would be performed by Bonferroni. The rates would be compared by the Chi-square test. If the statistical data did not conform to a normal distribution, a non-parametric Mann-Whitney U test would be used. Multivariate logistic regression analysis would be used to evaluate the effects of different treatments on the ongoing pregnancy rate, and adjustments were made according to the age, BMI, AFC, basal FSH level, and AMH level of patients. Bilateral p<0.05 would be defined as a significant difference.

## 3 Discussion

As far as we know, this is the first RCT to assess the impact of GH on IVF/ICSI outcomes and endometrial receptivity of patients undergoing GnRH-ant protocol with fresh embryo transfer. 120 participants would be recruited for analyzing the meaning of GH supplementation in improving the pregnancy outcomes and endometrial receptivity of IVF/ICSI patients undergoing GnRH-ant protocol with fresh embryo transfer. The primary outcome in this study is the ongoing pregnancy rate. And the secondary outcomes in this study include the Gn dosage, duration of COH, endometrial thickness and pattern, LH, E2, P level on trigger day, numbers of retrieved oocytes, high-quality embryo rate, biochemical pregnancy rate, clinical pregnancy rate, implantation rate, ectopic pregnancy rate, early miscarriage rate, multiple pregnancy rate and incidence of moderate and severe OHSS.

The pregnancy outcomes of fresh embryo transfer with GnRH-ant protocol are poor and affect the prognosis of patients. How to promote the outcomes of those patients has become a challenge in current clinical practice. A previous study of our department found that patients receiving GnRH-ant protocol for COH had the abnormal expression of multiple endometrial receptivity molecular markers, indicating abnormal endometrial receptivity compared with patients with depot GnRH-a protocol and natural cycle protocol (28). Another study found that GnRH antagonist can inhibit the expression of the c-kit receptor in endometrial stromal cells, thereby affecting the proliferation of stromal cells, and ultimately leading to a decrease in the embryo implantation rate and clinical pregnancy rate of patients receiving GnRH-ant protocol (29). Zhang D et al. found that the expression of endometrial apoptosis-related molecules was significantly increased in patients receiving the GnRH-ant protocol, and the expression level of S100P protein in endometrial epithelial cells was significantly decreased, which affected the endometrial receptivity (24). These findings suggest that GnRH antagonists may interfere with the normal function of the endometrium, affect the establishment of endometrial receptivity, and lead to the displacement of the window of implantation (30). To summarize the relevant mechanism, it may be related to the abnormal pathways of endometrial angiogenesis, cell proliferation/migration/apoptosis caused by GnRH antagonists (31).

Although GH has been applied in the field of ART for many years, the existing studies are mostly limited to improving the quality of oocytes and embryos in elderly patients and patients with diminished ovarian reserve, and there are few studies on improving endometrial receptivity. Classical studies have shown that there is no obvious expression of GH in the proliferative stage, but the expression of GH increases significantly in the mid-secretory glandular epithelium, suggesting that GH may play an integral role in the establishment of endometrial receptivity. GH receptors are widely expressed in endometrial stromal and epithelial cells (18, 32). Recent reviews have shown that GH can directly interact with endometrial GH receptors or indirectly mediated by insulin-like growth factor-1 (IGF-1), and improve endometrial thickness and sub-endometrial blood flow by improving the endometrial angiogenesis pathway (such as VEGF, etc.) and the expression of epithelial adhesion-related molecules (such as Integrinβ3, etc.), thus increasing the implantation rate and clinical pregnancy rate (32). To further verify these conclusions, we designed this prospective pilot study.

We assume that GH supplementation would promote the pregnancy outcomes and endometrial receptivity of IVF/ICSI patients undergoing GnRH-ant protocol with fresh embryo transfer. Understanding the association between GH and pregnancy outcomes and endometrial receptivity could provide solid evidence for clinical practice. If our hypothesis is confirmed, future studies will further explore the significance of GH in improving endometrial receptivity in certain populations such as patients with RIF, and multi-center RCTs with larger samples will be conducted to further investigate the optimal dosage and duration of GH in improving pregnancy outcomes in patients receiving fresh embryo transfer with COH through GnRH-ant protocol to further improve ART outcomes.

### 3.1 Strengths and limitations

Our study has several strengths. First, our RCT design could lead us to excavate the association between GH and pregnancy outcomes and endometrial receptivity and provide solid evidence for exploring the possible mechanisms. The GnRH-ant protocol is widely used in the world. Our results would provide a feasible method to solve the problem of poor pregnancy outcomes in patients receiving fresh embryo transfer with COH through GnRH-ant protocol and provide a basis for updating ART guidelines. Second, this RCT study designed by a multidisciplinary team of reproductive medicine clinicians, physicians, sonographers, statisticians, scientists, and other related experts, and multidisciplinary collaboration will guarantee the quality of research.

The main limitation of this study is the single-center study design in Hubei Province, China, without other reproductive medicine centers participation. In addition, the sample size is relatively small because it is a pilot study. Therefore, we will further confirm our conclusion by supplementing the results of relevant *in vitro* research.

## Ethics statement

The studies involving humans were approved by the Ethics Committee of Tongji Hospital, Tongji Medical College, Huazhong University of Science and Technology. The studies were conducted in accordance with the local legislation and institutional requirements. The participants provided their written informed consent to participate in this study.

## Author contributions

JY conceived and directed this study, and JY is the leading corresponding author. YC, SY, SL and DH were responsible for protocol writing and revising and recruiting patients. YC, LW and JX were responsible for sample size calculation, follow-up, endometrium collection and statistical analysis. YC and LW were involved in manuscript editing. All authors participated in the project and the acquisition, analysis, or interpretation of the data, and the final version has been reviewed and approved.
